# Guggulsterone inhibits prostate cancer growth via inactivation of Akt regulated by ATP citrate lyase signaling

**DOI:** 10.18632/oncotarget.6005

**Published:** 2015-10-05

**Authors:** Yajuan Gao, Yan Zeng, Jian Tian, Mohammad Shyful Islam, Guoqin Jiang, Dong Xiao

Published in Oncotarget, Advance publications. This article has been retracted due to falsified data in Figures 1,4,5,S2 and S3 reporting tumor experiments on animals.

The authors sincerely apologize to the scientific community for any confusion or adverse consequences resulting from the publication of this data.

http://www.impactjournals.com/oncotarget/index.php?journal=oncotarget&page=article&op=view&path[]=2138&author-preview=1ne

